# The Influence of Stored Energy on Grain Boundary Chemistry and Intergranular Corrosion Development in AA2024-T3 Alloy

**DOI:** 10.3390/ma11112299

**Published:** 2018-11-16

**Authors:** Xinxin Zhang, Xiaorong Zhou, Guangyi Cai, Yang Yu, Xueqin Lu, Yanbin Jiao, Zehua Dong

**Affiliations:** 1 Hubei Key Laboratory of Material Chemistry and Service Failure, School of Chemistry and Chemical Engineering, Huazhong University of Science and Technology, Wuhan 430074, China; guangyicai@hust.edu.cn (G.C.); yuyang@hust.edu.cn (Y.Y.); luxueqin@hust.edu.cn (X.L.); yanbinjiao@hust.edu.cn (Y.J.); 2 Corrosion and Protection Centre, School of Materials, The University of Manchester, Manchester M13 9PL, UK

**Keywords:** aluminum, electron microscopy, intergranular corrosion

## Abstract

Following our previous research, the correlation between the micro-chemistry of grain boundary and the distribution of stored energy in AA2024-T3 alloy is investigated, using the combination of transmission Kikuchi diffraction and transmission electron microscopy. It is found that the difference of dislocation density, namely stored energy, between two neighboring grains significantly affects the micro-chemistry of the grain boundary. Further, it is revealed that intergranular corrosion development in the AA2024-T3 alloy is mainly attributed to the combined effect of grain boundary chemistry and stored energy distribution.

## 1. Introduction

AA2024 alloy has been extensively utilized in the transportation industry as a high strength aluminium alloy. However, it is not immune to intergranular corrosion in a chloride-containing environment [1,2,3,4]. Even though a number of surface engineering methods have been applied to increase the corrosion resistance, as well as other surface properties (hardness and wear resistance) of aluminium alloys [5,6,7], intergranular corrosion still occurs in AA2024 alloy. Thus, to figure out the root cause for intergranular corrosion of aluminium alloy is necessary, which may provide the crucial theoretical guidance for surface engineering and other corrosion protection methods. Hence, numerous studies have been conducted, to study the intergranular corrosion mechanism of aluminium alloy [1,8,9,10].

Previous work has focused on the effect of grain boundary micro-chemistry on intergranular corrosion. It is pointed out that the chemical heterogeneity at the grain boundary results in the formation of galvanic coupling, which leads to the selective dissolution of the more anodic part and, thus, leads to intergranular corrosion development [4,11,12,13,14]. For AA2024 alloy, it is revealed that the distribution of elemental Cu significantly affects intergranular corrosion development. Due to the more positive electrode potential of Cu relative to Al, Cu-rich grain boundary precipitates [1,10,15,16,17,18], Cu-enriched layers [19,20,21] and Cu segregation [12,22,23] at the grain boundary act as effective cathodes to promote the anodic dissolution of aluminium in the periphery, which consequently results in intergranular corrosion in the alloy.

However, a more recent work has shown that intergranular attack could occur at the grain boundary in the absence of compositional heterogeneity [24]. AA2024 alloy is usually supplied in the T3 condition. The T3 thermomechanical process of aluminum alloys typically involves three steps, namely solution heat treatment, cold working, and final natural ageing to a substantially stable condition. Since natural ageing is conducted at room temperature instead of at elevated temperatures, the precipitation process is significantly retarded in the alloy of the T3 condition, which may result in a limited chemical heterogeneity of the alloy. By contrast, due to the presence of cold working, a high population density of dislocations is always introduced into the alloy in the T3 condition, resulting in a non-uniform distribution of dislocations. Hence, it is believed that, in addition to chemical heterogeneity, structural heterogeneity also affects the intergranular corrosion development [4,23,25,26,27,28]. In our previous work, it is revealed that the distribution of grain-stored energy, namely dislocation density, significantly affects the intergranular corrosion development of aluminium alloys [21,23,29,30,31,32,33,34]. During immersion in NaCl solution, intergranular corrosion preferentially occurs around grain with a higher stored energy rather than that of lower stored energy [21,23,31]. The intergranular corrosion is not confined to the grain boundary, which selectively develops into the interior of the neighboring grain of high stored energy with prolonged immersion time [23,31,35].

**Figure 1:**
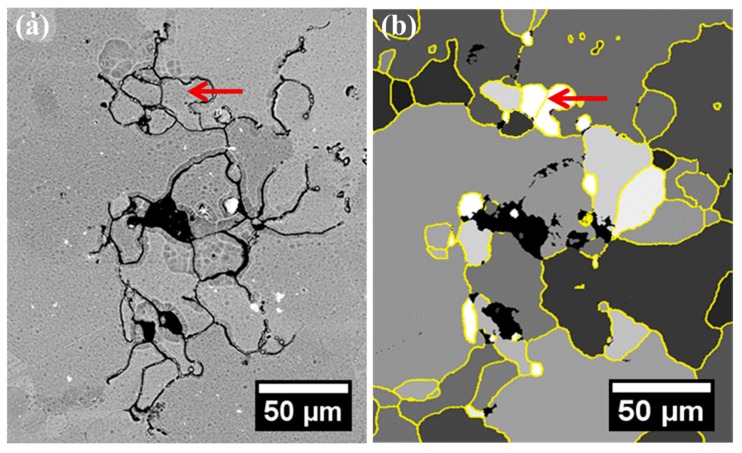
(**a**) SEM micrograph of a typical localized corrosion site after 10 h immersion in a 3.5 wt % NaCl solution; (**b**) the corresponding grain-stored energy distribution in grey scale, with the yellow lines representing grain boundaries.

As revealed in our previous work, the correlation between intergranular corrosion and stored energy distribution is highly reliable and reproducible [20,21,31,35] with a typical example being shown in Figure 1. Figure 1a displays a scanning electron micrograph (SEM, FEI, Hillsboro, OR, USA) of a localized corrosion site on an alloy surface after immersion in a 3.5 wt % NaCl solution for 10 h. A surface texture is present, since the plasma cleaning is carried out to attain a stress-free surface prior to SEM observation. Scrutiny of Figure 1a reveals that dark, narrow lines, namely the attacked grain boundaries, distribute non-uniformly on the alloy surface, suggesting different corrosion susceptibilities of the grain boundaries.

The corresponding stored energy distribution, which represents the dislocation density, is displayed in Figure 1b, with yellow lines representing the grain boundaries. The calculation procedure of stored energy distribution is as follows. During electron backscatter diffraction (EBSD) mapping (Oxford Instrument, Oxford, UK), the orientation of each pixel was obtained. For a neighboring pixel pair with an orientation difference that is higher than a threshold value of 1°, the stored energy could be obtained using the Read–Shockley equation shown as following:

γ_s_ = γ_0_ θ (A − ln θ)

where θ = b/h, γ_0_ = Gb/4π(1 − ν), A = 1 + In(b/2πr_0_), G is the shear modulus, ν is Poisson’s ratio, and r_0_ is the radius of the dislocation core. Then, the stored energy of an individual grain could be determined by averaging the sum of the energy of all pixel pairs within the grain. Finally, the spatial distribution of the grain-stored energy could be determined, which is then displayed as a stored energy map in grey-scale with the low brightness representing low stored energy, and vice versa [21,31,35].

Comparing the distribution of grain-stored energy (Figure 1b) to the corrosion morphology (Figure 1a), it is obvious that the attacked grain boundaries are selectively located in the periphery of grain with high stored energy, instead of that with low stored energy, consistent with our previous work [21,23,31].

However, an exception is also noticed in Figure 1, as marked with a red arrow. Evidently, the boundary marked by the arrow locates in the periphery of the grains of high levels of stored energy, but it is still intact after immersion testing. The unexpected observation suggests that even though the stored energy distribution plays a decisive role during intergranular corrosion development [21], other factors also affect the corrosion process, which deserves further investigation. Herein, the influence of stored energy on the micro-chemistry of grain boundary was investigated in AA2024-T3 alloy, in order to have a better understanding of the correlation between grain-stored energy distribution and intergranular corrosion.

## 2. Experimental Methods

AA2024-T3 alloy (Cu 4.65 wt %; Fe 0.21 wt %; Mg 1.54 wt %; Mn 0.52 wt %; Si 0.088 wt %; Zn 0.11 wt % ; Al remaining) was investigated in this work. The specimens with the dimensions of 15 mm × 20 mm were prepared from a cold rolled sheet of 1.2 mm thickness. The specimens were successively ground with 800, 1200, 2500, and 4000 grit silicon carbide papers, polished sequentially using 3 µm and 1 µm diamond pastes, and finalized with an OP-S polishing treatment to ensure the surface roughness Ra was lower than 0.1 μm. After mechanical polishing, the specimens with surface hardness ranging from 123.6 HB to 125.9 HB were cleaned ultrasonically in an acetone bath and dried in a cool air stream.

Corrosion immersion testing was carried out by immersing mechanically-polished specimens in a 3.5 wt % NaCl solution at an ambient temperature for 10 h with at least five samples being tested to ensure the repeatability of the results. After the immersion testing, the specimen was thoroughly rinsed with deionized water and dried in a cool air stream. After the immersion testing, glow discharge optical emission spectroscopy equipment was employed for 30 s at an argon pressure of 750 Pa and power of 15 W, to obtain contamination- and strain-free surfaces for SEM observation and EBSD analysis.

Twin-jet electropolishing of the alloy was carried out using a mixture of 700 mL methanol and 300 mL nitric acid at a temperature of −20 °C, to prepare the electron transparent foils for transmission electron microscopy (TEM, FEI, Hillsboro, OR, USA). Focused ion beam (FIB) was employed to obtain the cross-sections of both the as-received alloys and stable localized corrosion sites after corrosion immersion testing for TEM observation, transmission Kikuchi diffraction (TKD, Oxford Instrument, Oxford, UK), and energy dispersive X-ray (EDX, Oxford Instrument, Oxford, UK) analysis.

## 3. Results

**Figure 2:**
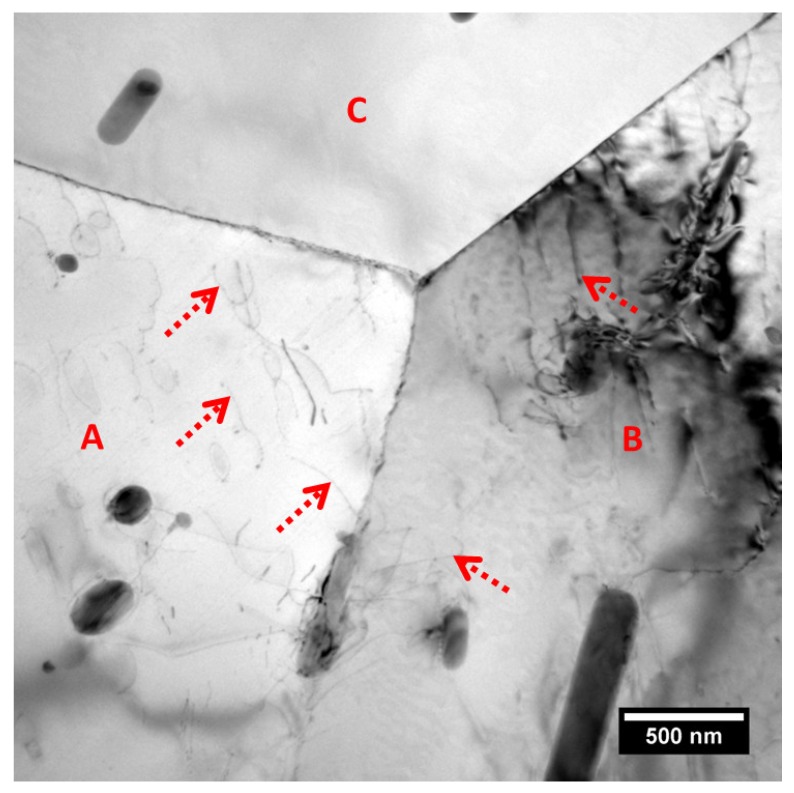
Transmission electron microscopy (TEM) micrograph of a typical grain boundary junction in AA2024-T3 alloy.

A typical grain boundary junction in AA2024-T3 alloy is presented in a bright field TEM micrograph (Figure 2), exhibiting three typical grain boundaries of the alloy. Curved and narrow lines, which are supposed to be dislocations, are present as marked with dashed-line arrows. Evidently, higher population densities of dislocations exist in grains A-B rather than grain C, resulting in non-uniform distribution of dislocations across the alloy, and thus, a heterogeneous distribution of grain-stored energy, which is consistent with Figure 1. Correspondingly, the distribution of the grain boundary precipitates is also non-uniform with the presence of precipitates only at boundary A-B rather than boundaries A-C and B-C, indicating the possible relationship between the stored energy distribution and the micro-chemistry of the grain boundary.

**Figure 3:**
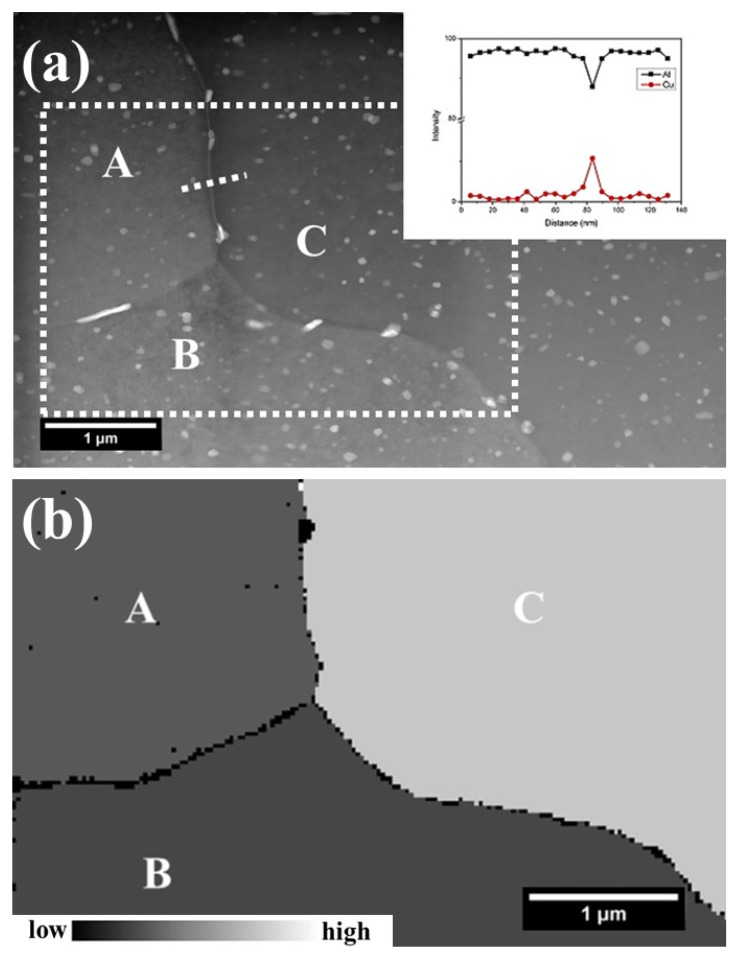

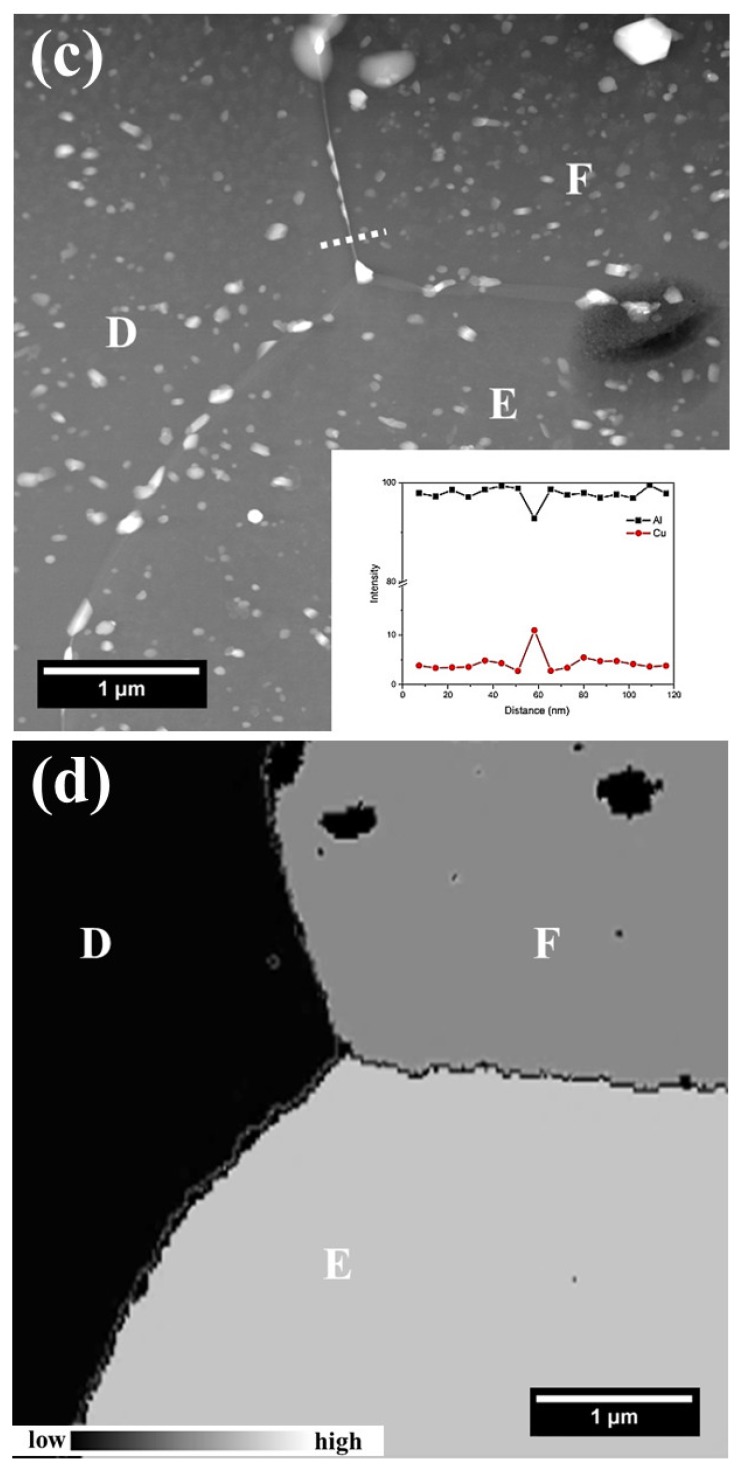
(**a**) High angle annular dark field (HAADF) micrograph of typical grain boundaries in AA2024-T3 alloy with the elemental profile along the dashed line inset; (**b**) stored energy distribution of the framed area in (**a**); (**c**) HAADF micrograph of typical grain boundaries in the AA2024-T3 alloy; (**d**) the corresponding stored energy distribution of (**c**).

In order to examine the correlation between stored energy and grain boundary chemistry, both TEM and TKD analyses were conducted on the typical areas of AA2024-T3 alloy, which were generated using FIB. Figure 3a exhibits a high angle annular dark field (HAADF) micrograph of a typical area in the alloy, which contains three grains, A, B, and C, along with three grain boundaries A-B, A-C and B-C. Bright features of nanometer scales, namely θ’ (Al_2_Cu) phase precipitates [19,33,34], were present at all three boundaries, even though the population density varied significantly. At boundary A-B, only one coarse precipitate was present, whereas much higher population densities of fine precipitates existed at boundary B-C. In addition to the fine precipitates, the segregation of Cu was also observed at boundary A-C, as revealed by an EDX line-scan with an elemental profile along a dashed-line inset. The grain-stored energy distribution corresponding to the framed area in Figure 3a is shown in Figure 3b, revealing that grains A-B contain lower levels of stored energy, whereas grain C has much higher stored energy.

Similarly, HAADF micrographs of another area in AA2024 alloy are shown in Figure 3c, exhibiting three grain boundaries D-E, D-F, and E-F. Similar with Figure 3a, Figure 3c reveals the presence of precipitates with typical sizes ranging from tens of nanometers to over one hundred nanometers at grain boundaries. A scrutiny of Figure 3c reveals that much higher amounts of precipitates exist at boundaries D-E and D-F relative to the grain boundary E-F. Along with precipitates, Cu segregation was also detected along boundary D-F with the elemental profile along the dashed line inset in Figure 3c, whereas no segregation was detected at boundary E-F. The corresponding distribution of grain-stored energy is shown in Figure 3d, revealing that both grains E-F contained much higher levels of stored energy relative to grain D.

Comparing grain boundary chemistry (Figure 3a,c) with the corresponding stored energy distribution (Figure 3b,d), it is revealed that when both neighboring grains contain similar levels of stored energy (regardless of high or low level), a lower population density of precipitates is present at the grain boundary (boundaries A-B from Figure 3a and E-F from Figure 3c), whereas much higher population densities of precipitates were observed along the boundary between grains with evidently different levels of stored energy, which may also be decorated with Cu segregation (boundaries A-C, B-C from Figure 3a and D-E, D-F from Figure 3c). As a result, a nearly continuous galvanic coupling could form at the boundary, with neighboring grains containing evidently different levels of stored energy. By contrast, a relatively low continuity of galvanic coupling is noticed at boundaries when neighboring grains contain similar levels of stored energy. Hence, it is evident that the stored energy distribution significantly affects the micro-chemistry of the grain boundary.

**Figure 4:**
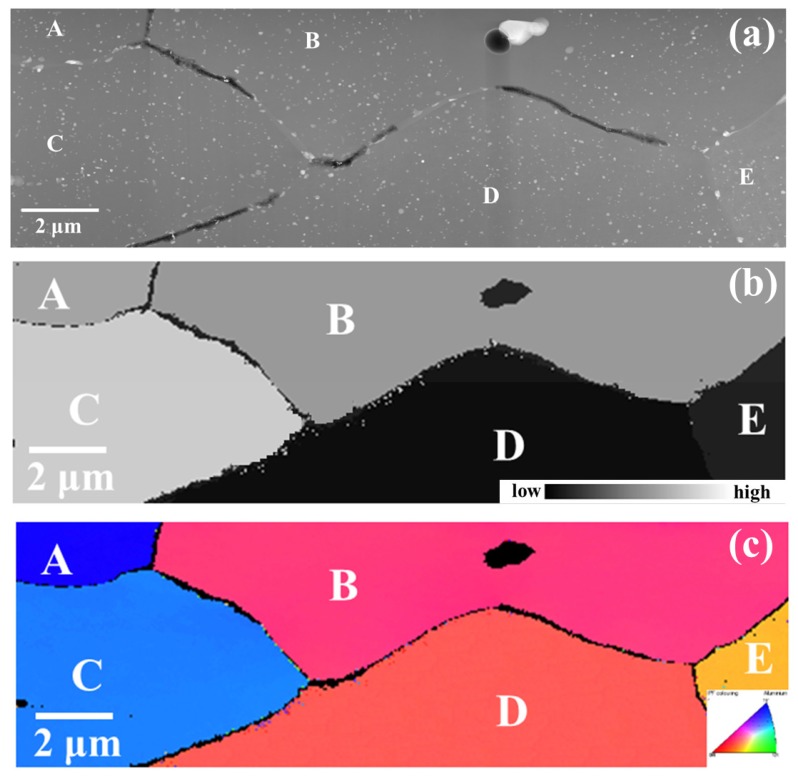
(**a**) HAADF micrograph of the cross-section of a typical localized corrosion site in AA2024-T3 alloy after 10 h immersion in a 3.5 wt % NaCl solution; (**b**,**c**) the corresponding distributions of stored energy in grey scale and grain orientation in IPF colouring.

To correlate intergranular corrosion to the local grain structure and the grain boundary chemistry, both TEM and TKD were conducted at the same site. Figure 4a displays the HAADF micrograph of a cross-section at localized corrosion site after immersion in a 3.5 wt % NaCl solution for 10 h. The presence of dark and narrow lines, namely attacked boundaries, suggests the occurrence of intergranular corrosion. During the immersion in NaCl solution, the electrolyte providing the necessary chemical condition accesses the sub-surface area via attacked grain boundaries. Thus, grain boundaries near the surface are exposed to the aggressive medium longer than those away from the surface. However, attacked grain boundaries are present at various depths beneath the alloy surface, suggesting an intergranular corrosion susceptibility difference of grain boundaries. The corresponding distributions of stored energy in greyscale and grain orientation in inverse pole figure (IPF) coloring are shown in Figure 4b,c. It is evident that grain-stored energy distributes non-uniformly, which follows a decreasing order: grain C > grain A ≈ grain B > grain E > grain D. Comparing the stored energy distribution (Figure 4b) to the corrosion morphology (Figure 4a), it is revealed that a boundary attack tends to occur around grains with high levels of stored energy, rather than those with low levels of stored energy, consistent with previous work [21,23,31]. Unexpectedly, grain boundary A-C that exists at the near-surface area with both neighboring grains containing high levels of stored energy remained intact after 10 h immersion, revealing that in addition to stored energy, other factors also affect the intergranular corrosion, consistent with Figure 1.

**Figure 5:**
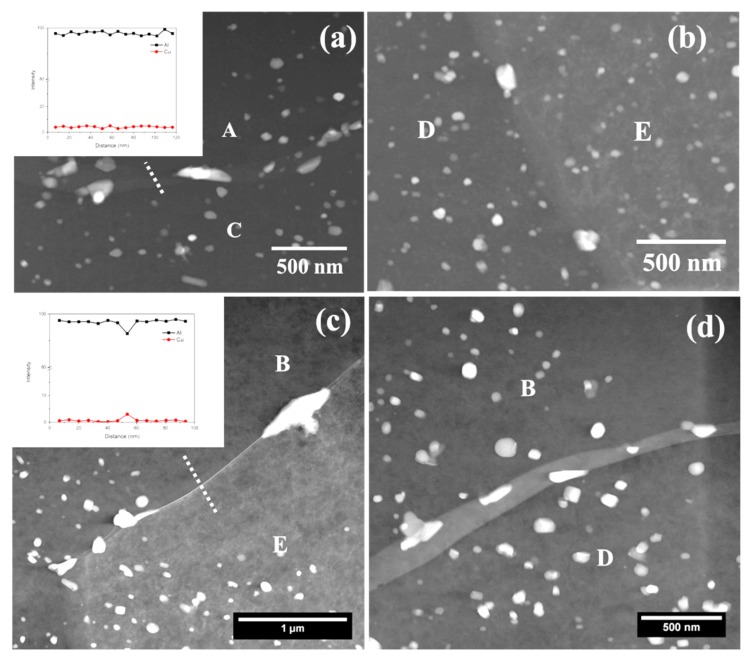
(**a**) HAADF micrograph of boundary A-C of Figure 4 with elemental profile inset; (**b**) HAADF micrograph of boundary D-E of Figure 4; (**c**) HAADF micrograph of boundary B-E of Figure 4 with elemental profile inset; (**d**) HAADF micrograph of boundary B-D of Figure 4.

To figure out the root cause for the high intergranular corrosion resistance of grain boundary A-C, it was then examined using TEM. Figure 5a displays the grain boundary A-C at increased magnification, displaying the presence of only a few individual precipitates at the boundary. Further, EDX analysis reveals the absence of segregation at boundary A-C with the corresponding elemental profile along the dashed line inset. Hence, the continuity of galvanic coupling along the grain boundary A-C is interrupted, which may lead to its high intergranular corrosion resistance [8,9,10,35]. In addition to boundary A-C, the micro-chemistry of other boundaries in the area of Figure 4 was also examined. Figure 5b displays the HAADF micrograph of grain boundary D-E, with both neighboring grains showing low levels of stored energy, exhibiting the presence of only a few precipitates at the boundary. By contrast, grain boundary B-E and B-D with one neighboring grain of relatively high stored energy and the other one of relatively low stored energy are shown in Figure 5c,d at increased magnifications, revealing the presence of high amounts of grain boundary precipitates at both boundaries. Further, the elemental profile along the dashed line in Figure 5c is inset, indicating that, in addition to the precipitates, Cu segregation is also present at the boundary. Thus, consistent with Figure 3, grain boundary chemistry is closely related to the corresponding stored energy distribution. When neighboring grains contain similar stored energy, the grain boundary decoration is less continuous, resulting in the low continuity of galvanic coupling at grain boundaries, whereas a nearly continuous galvanic coupling along the grain boundary could be formed when the stored energy difference of the neighboring grains is significant, due to the presence of both precipitates and segregation at the boundary.

**Figure 6:**
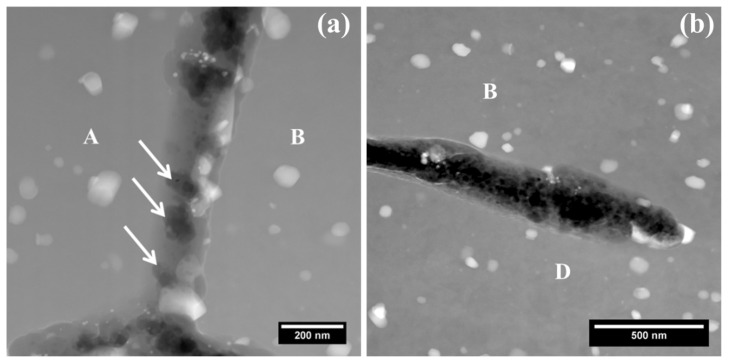
(**a**) HAADF micrograph of boundary A-B of Figure 4; (**b**) HAADF micrograph of boundary B-D of Figure 4.

Finally, attacked grain boundaries at the site of Figure 4 were examined with two typical examples shown in Figure 6a,b. Evidently, both of the attacked grain boundaries exhibited hundreds of nanometers of width, revealing that intergranular corrosion is confined to the grain boundary itself, but it further develops into the interiors of neighboring grain interiors [1,20,21,35]. Figure 6a displays the attacked grain boundary A-B, exhibiting the presence of cavities as marked with solid-line arrows, which have similar dimensions and shapes of the grain boundary precipitates (Figure 3). Thus, it is suggested that intergranular attack at the boundary A-B is closely associated with the dissolution of individual precipitates instead of the continuous galvanic coupling along the boundary [1,21,23]. Hence, the presence of individual cavities at the boundary indicates the low continuity of galvanic coupling along the boundary, corresponding to similar levels of stored energy between grains A and B (Figure 4 and Figure 5). 

Further, Figure 6b displays the attacked grain boundary B-D, which exhibits an intergranular corrosion filament with the absence of individual cavities at the boundary, compared to boundary A-B. High concentration of precipitates along with Cu segregation at the boundary (Figure 5d) results in continuous galvanic coupling, which results in the continuous dissolution of aluminium and, consequently, leads to the formation of intergranular corrosion filaments with smooth walls, rather than individual cavities [1,3]. Therefore, from Figure 4, Figure 5 and Figure 6, it is believed that the combined effect of stored energy and grain boundary chemistry affects the intergranular corrosion development in AA2024-T3 alloy.

## 4. Discussion

### 4.1. Microstructural Evolution

The combination of TEM and TKD analysis successfully correlates stored energy distribution with grain boundary chemistry (Figure 3). It is found that when neighboring grains contain significantly different levels of stored energy, a more continuous galvanic coupling at the boundary could be formed, whereas the continuity of galvanic coupling was interrupted at the boundary with neighboring grains containing similar levels of stored energy (Figure 3, Figure 4 and Figure 5). 

It is believed that the correlation between the micro-chemistry of the grain boundary and grain-stored energy distribution is closely associated with its thermomechanical history [36,37,38]. The as-received AA2024-T3 alloy was supplied as a sheet after cold rolling. During the rolling, a large amount of dislocations was generated in the alloy. The generation of dislocation is grain orientation-dependent during the cold working process, since the number of active slip systems within the grain highly depends on the orientation relationship between the grain orientation and the fabrication direction. A higher population density of dislocations could be generated in the grains with more active slip systems and vice versa, which leads to the heterogeneous distribution of dislocations, namely stored energy, within different grains [39]. 

Following the sheet formation, the solution heat treatment was then conducted at over 500 °C, which promotes the diffusion of solute atoms due to the high temperature. Hence, during the solution heat treatment, grains with high levels of stored energy contain high amounts of dislocations, which provide a fast diffusion path for solute atoms and thus results in an increased amount of solute atoms [22]. Meanwhile, the clustering of vacancy and solute atoms results in the formation of vacancy–solute atom pairs, which tend to diffuse to the grain boundary, driven by the concentration gradient. As a result, the solute atoms segregate at the grain boundary with the annihilation of the vacancy [40]. Hence, with higher amounts of dislocations within the neighboring grains, an increased amount of solute atoms are segregated at the grain boundary. Therefore, when both neighboring grains contain higher levels of stored energy, namely dislocation densities, the higher amount of solute atoms is segregated at the corresponding grain boundary, and vice versa.

Then, during the following natural ageing process, grain boundary precipitation occurs, which is mainly driven by the saturated contents of the solute atoms at the grain boundaries. Depending on the amount of grain boundary segregation, the micro-chemistry of the grain boundary changes accordingly. For a grain boundary that locates between two grains with high levels of stored energy, numerous precipitates form, with a significant level of consumption of solute atoms, which finally leads to the absence of segregation (Figure 3 and Figure 5). By contrast, a low amount of solute atom segregation at the grain boundary results in the formation of only a few precipitates (Figure 3 and Figure 5). For the grain boundary locating between grains of significantly different levels of stored energy, relatively low amounts of nucleation sites exist at the grain boundary, which consequently leads to a lower population density of precipitates compared to that locating between both grains of high levels of stored energy (Figure 3 and Figure 5). Meanwhile, the solute atoms that were not consumed by precipitation could remain at the grain boundary, resulting in grain boundary segregation (Figure 3 and Figure 5). The precipitates, along with segregation at the boundary, finally result in continuous galvanic coupling between the grain boundary and the matrix in the periphery, which may increase its intergranular corrosion susceptibility significantly, as discussed in the following section (Figure 4, Figure 5 and Figure 6).

### 4.2. Intergranular Corrosion

In our previous work, the influence of stored energy distribution on intergranular corrosion development is discussed [21,23,24,31,41]. It is presumed that high levels of stored energy lead to high thermodynamic instability, resulting in a high corrosion susceptibility. As a result, the intergranular corrosion susceptibility of the grain boundary in the periphery of grain with high stored energy is higher than that around the grain of low stored energy. However, in the present work, it is revealed that the boundary with both neighboring grains containing high levels of stored energy may exhibit lower intergranular corrosion susceptibility, compared to that with the neighboring grains containing significantly different levels of stored energy (Figure 1 and Figure 4).

When the alloy was immersed in the NaCl solution, the development of intergranular corrosion was determined by the combined effect of the stored energy and the grain boundary chemistry. Intergranular corrosion, which is not confined to the grain boundary itself, involves the anodic dissolution of the grain boundary itself, as well as the interior of the neighboring grains [21,23,31]. It is well-documented that the selective dissolution of the grain boundary itself is closely associated with the chemistry of the grain boundary, especially segregations and precipitates at the grain boundary [11,12,13,14,21]. Hence, for grain boundaries with both neighboring grains containing high levels of stored energy, a low continuity of galvanic coupling may lead to its increased intergranular corrosion resistance relative to that with neighboring grains containing significantly different levels of stored energy (Figure 1 and Figure 4). 

After the dissolution of the grain boundary itself, the following process involves the dissolution of the matrix in its periphery, which is mainly dependent on the stored energy. The higher level of stored energy of the grain could result in its increased thermodynamic instability, and thus, a higher corrosion susceptibility. As a result, intergranular corrosion tends to further develop by dissolving the neighboring grains with high levels of stored energy [21,23]. Hence, the grain boundary in the periphery of the grain with high stored energy generally exhibits high intergranular corrosion susceptibility.

## 5. Conclusions

The present work examines the correlation between stored energy distribution and grain boundary chemistry. It is revealed that when both neighboring grains contain high levels of stored energy, a high density of precipitates is present at the boundary, whereas a much lower density of grain boundary precipitates is formed at the grain boundary with both neighboring grains showing low levels of stored energy. When the stored energy of the neighboring grains exhibits significant differences, in addition to precipitates, Cu segregation may also exist at the grain boundary. 

Intergranular corrosion development in AA2024-T3 alloy is closely associated with the combined effect of stored energy distribution and grain boundary micro-chemistry. It is revealed that with a significant difference in stored energy between neighboring grains, in addition to precipitates, Cu segregation may also exist at the grain boundary, which could form a continuous galvanic coupling with the matrix and thus result in higher intergranular corrosion susceptibility, in comparison to boundaries locating between grains of similar levels of stored energy.
